# Genomic survey of the ectoparasitic mite *Varroa destructor*, a major pest of the honey bee *Apis mellifera*

**DOI:** 10.1186/1471-2164-11-602

**Published:** 2010-10-25

**Authors:** Scott R Cornman, Michael C Schatz, Spencer J Johnston, Yan-Ping Chen, Jeff Pettis, Greg Hunt, Lanie Bourgeois, Chris Elsik, Denis Anderson, Christina M Grozinger, Jay D Evans

**Affiliations:** 1 USDA-ARS, Bee Research Laboratory, 10300 Baltimore Ave., Beltsville, MD 20705 USA; 2 Center for Bioinformatics and Computational Biology, University of Maryland, College Park, MD 20742 USA; 3 Department of Entomology, Texas A&M University, College Station, TX 77843 USA; 4 Department of Entomology, Purdue University, West Lafayette, IN 47907 USA; 5 USDA-ARS, Honey Bee Research Laboratory, 1157 Ben Hur Rd., Baton Rouge, LA 70820 USA; 6 Department of Biology, Georgetown University, 37th and O Streets, NW, Washington, DC 20057 USA; 7 CSIRO Entomology, Black Mountain Laboratories, Clunies Ross Street, Black Mountain ACT 2601, Australia; 8 Department of Entomology, Penn State University, University Park, PA 16802 USA

## Abstract

**Background:**

The ectoparasitic mite *Varroa destructor *has emerged as the primary pest of domestic honey bees (*Apis mellifera*). Here we present an initial survey of the *V. destructor *genome carried out to advance our understanding of *Varroa *biology and to identify new avenues for mite control. This sequence survey provides immediate resources for molecular and population-genetic analyses of *Varroa*-*Apis *interactions and defines the challenges ahead for a comprehensive *Varroa *genome project.

**Results:**

The genome size was estimated by flow cytometry to be 565 Mbp, larger than most sequenced insects but modest relative to some other Acari. Genomic DNA pooled from ~1,000 mites was sequenced to 4.3× coverage with 454 pyrosequencing. The 2.4 Gbp of sequencing reads were assembled into 184,094 contigs with an N50 of 2,262 bp, totaling 294 Mbp of sequence after filtering. Genic sequences with homology to other eukaryotic genomes were identified on 13,031 of these contigs, totaling 31.3 Mbp. Alignment of protein sequence blocks conserved among *V. destructor *and four other arthropod genomes indicated a higher level of sequence divergence within this mite lineage relative to the tick *Ixodes scapularis*. A number of microbes potentially associated with *V. destructor *were identified in the sequence survey, including ~300 Kbp of sequence deriving from one or more bacterial species of the Actinomycetales. The presence of this bacterium was confirmed in individual mites by PCR assay, but varied significantly by age and sex of mites. Fragments of a novel virus related to the Baculoviridae were also identified in the survey. The rate of single nucleotide polymorphisms (SNPs) in the pooled mites was estimated to be 6.2 × 10^-5^per bp, a low rate consistent with the historical demography and life history of the species.

**Conclusions:**

This survey has provided general tools for the research community and novel directions for investigating the biology and control of *Varroa *mites. Ongoing development of *Varroa *genomic resources will be a boon for comparative genomics of under-represented arthropods, and will further enhance the honey bee and its associated pathogens as a model system for studying host-pathogen interactions.

## Background

Honey bees (*Apis mellifera*) are an important agricultural commodity providing honey, other bee products, and pollination services [1,2]. Domesticated honey bees in the United States and elsewhere have been in decline in recent years, despite an increasing need for honey bee pollination services [3]. This fact is often blamed on increasing challenges from pests and pathogens, as well as episodes of severe decline such as the enigmatic 'colony collapse disorder' (CCD) [4].

Among the most detrimental of honey bee pests is the ectoparasitic mite *Varroa destructor *[5]. *V. destructor *and its closely related congener, *V. jacobsoni*, are native to Asia where they parasitize the Eastern honey bee, *A. cerana. V. destructor *was only identified as a morphologically and genetically distinct species from *V. jacobsoni *relatively recently [6]. *V. destructor *began to appear in Asian colonies of *A. mellifera *during the last century and is now widely distributed, inadvertently aided by trade in bees and bee products.

Mite-infested bee colonies suffer directly from parasitism of pupae and adults, and indirectly from viral and microbial pathogens that the mites vector [7,8]. Feeding by mites induces an immunosupression in bees that leads to increased titres of pre-existing infections [9], further compounding their impact. The economic toll of *V. destructor *on apiculture is estimated to be millions of U.S. dollars per year, and chemical control agents are worrisome both for their collateral effects on bee health and the potential for honey contamination [10].

*Varroa*-honey bee interactions are mediated to a large extent via chemical cues, and bees have numerous mechanisms to control *Varroa *populations (reviewed in [5,11]). *Varroa *mites reproduce on honey bee pupae, using chemical signals produced by the developing honey bee larvae to target appropriately aged hosts. The mature female offspring of reproductive *Varroa *emerge with the adult honey bee, and subsequently move to nurse bees (which are engaged in brood care), thereby allowing them to remain in close proximity to the brood [12,13]. Honey bees resist 'Varroatosis', the infestation of colonies by *Varroa *mites, via grooming of adult infested bees, removal of infested pupae (hygienic behavior), and physiological resistance mechanisms [5]. Recent successes in breeding *Varroa*-resistant bees, including the selection of 'Russian' bees with longstanding exposure to mites [14,15], indicate that a better understanding of how bees and mites interact with each other can lead to novel management strategies.

Comparative studies of the fragility of the *A. mellifera *- *V. destructor *interaction, which has apparently prevented most Asian lineages of *V. destructor *as well as other *Varroa *species from colonizing *A. mellifera *[6,16-18], supports the hypothesis that mite olfaction or other requirements for mite reproduction may be suitable control targets. A molecular-genetic approach to develop such innovative controls would clearly benefit from further insights into *Varroa *genomics, which could be exploited in conjunction with tools already extant for honey bee. Prior to this study, genes for only two non-mitochondrial *V. destructor *proteins had been deposited in GenBank, a sodium channel gene (AAN37408.1) and a glycoprotein (ACU30143.1). Genome sequencing will greatly expand this gene catalog, and may also uncover unforeseen targets for novel and specific acaricides, such as divergence in metabolic pathways between mites and bees or the discovery of important microbial interactions.

High-throughput, shotgun sequencing of whole genomes allows the rapid identification of thousands of genic sequences, greatly facilitating molecular and population-genetic studies that would otherwise proceed in piecemeal and laborious fashion. Here we report an initial sequence survey of the *V. destructor *genome in conjunction with a flow-cytometric estimate of genome size. Our annotations and analysis should aid investigators seeking molecular approaches to mite control. They will also provide a guide for a planned full genome project for this species [19], one of several genomics initiatives that are unfolding the molecular interactions between honey bees and a constellation of potentially interacting pathogens [4,7,20,21].

Of the eight genetically distinct lineages of *V. destructor *that parasitize *A. cerana *in Asia, two have been identified on *A. mellifera *[6,18,22,23]. Anderson [24] designated these lineages the Japan (J) and Korea (K) 'haplotypes' in reference to mitochondrial DNA makers, but they are concordantly distinct at nuclear markers as well [23]. Genetic differentiation within lineages is low [23], likely reflecting the population-genetic impact of life-history traits [5] such as full-sib mating and male haploidy [25], as well as potential population bottlenecks tied to host-shift events and subsequent range expansion [18,23]. In this study, we have analyzed the K haplotype of *V. destructor *from *A. mellifera*, the predominant haplotype presently found in North America [23]. We have identified over 13,000 contigs with sequences homologous to other species; many of these have recognized domains and/or functional annotations transferred from other arthropods. Interestingly, *V. destructor *appears to have experienced a higher rate of protein evolution than *Ixodes scapularis *since their divergence from the most recent common ancestor over 300 million years ago. Sequences attributable to a range of microbes were identified, including a large number of sequences from one or more novel actinomycete bacteria, the presence of which was confirmed by PCR in individual mites but not in adult honey bees. We also identified a novel virus related to the Baculoviridae that was abundant in the genomic survey. Finally, we found a low level of nucleotide polymorphism in the sequenced sample of ~1,000 mites, consistent with expectation [23]. This bodes well for future efforts to sequence and assemble a reference genome for this species and to identify genetic variation that correlates with host-interaction traits among *Varroa *strains and species.

## Results

### Genome size

**Figure 1:**
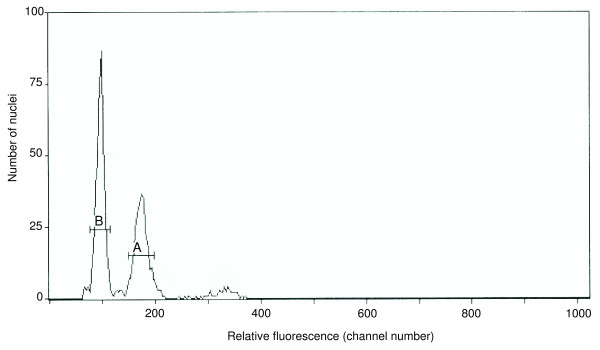
**2C genome size estimate for *Varroa destructor***. 2C genome size estimate for *V. destructor *based on flow cytometry, normalized to the *Drosophila virilis *genome (A). The *V. destructor *fluorescence peak (B) corresponds to a genome size of 0.577 picograms or 565 ± 3 Mbp.

Flow cytometry of *V. destructor *nuclei (normalized to nuclei of *Drosophila virilis*) yielded a haploid genome size estimate of 565 ± 3 Mbp (Figure 1). This genome size is larger than that of many insects but substantially smaller than that of numerous mites and ticks for which genome projects are underway or have been proposed (> 2,000 Mbp, [26]. It is also lower than the general range for ticks, which spans from roughly 1,000 Mbp in the argasid soft tick *Ornithodoros turicata *to 3,100 Mb in the ixodid hard tick *Amblyomma americanum *[27]. However, examples of very small acarid genomes are known. Most notably, the two-spotted spider mite *Tetranychus urticae*, which has been advocated as a model for genetic and developmental studies of chelicerates [28]), has an estimated genome size of only 75 Mbp. It is not yet clear to what extent these large differences in genome size are driven by variation in gene content, repetitive fraction, and/or ploidy. While the contributions of the latter two factors have been frequently noted, the potential contribution of gene expansion has been highlighted by recent analyses of waterflea [29] and pea aphid [30]that reveal a roughly two-fold increase in gene content relative to other arthropods.

### Sequencing, assembly, and filtering

Six pyrosequencing runs on a Genome Sequencer FLX instrument (454 Life Sciences) produced 2.4 Gbp of unpaired sequencing reads. After trimming low quality sequence, the average read length was 365 bp, generating an expected genome coverage of 4.2×. The reads were then assembled into contigs with the CABOG package of Celera Assembler version 5.2 [31]. The resulting assembly had a realized median contig coverage of 4.3×, but was highly fragmented and summed to only 318 Mbp of sequence, substantially less than the estimated genome size of 565 Mbp. Table 1 provides statistics for this assembly before and after removing problematic or undesired sequences (detailed below). The modest contig lengths are primarily a consequence of low coverage, as over 97% of contigs greater than 1 Kbp terminate due to coverage gaps rather than ambiguity from repetitive sequences. In fact, contig lengths were comparable to that expected (~3,860 bp) for an idealized assembly of equivalent coverage and read length, following the method of [32].

**Table 1:** Statistics of the *Varroa destructor *genome sequence survey

Statistic	Initial assembly	Filtered assembly
Number of contigs	271,543	184,094

Sum of contig length (Mbp)	318	294

Maximum contig length (bp)	18,703	16,332

Mean contig length (bp)	1,170.4	1,597.5

N50 contig length (bp)	2,107	2,262

Contigs ≥ 1,000 bp	107,195	105,621

Contigs ≥ 5,000 bp	5,407	5,374

Contigs ≥10,000 bp	120	118

Mean coverage	4.3	5.0

As detailed in the Methods, the small physical size of *V. destructor *required the collection of large numbers of mites from multiple honey bee colonies in order to obtain sufficient DNA for this survey. While mites were carefully cleaned and examined under a dissection microscope to remove any non-target organisms that might have been collected inadvertently, whole-organism extractions necessarily entail the possible inclusion of associated microbes, particularly gut microbes. The gut flora may include symbiotic, commensal, and pathogenic prokaryotes and eukaryotes, which are often important components of the ecology of arthropod species. We therefore filtered the assembled contigs based on G+C content, coverage, and sequence homology, in order to minimize the mis-annotation of microbial sequences as *V. destructor*, as well as to identify novel microbes of interest. Comparable strategies have been successfully applied to the classification of metagenomic samples (e.g., [33]).

**Figure 2:**
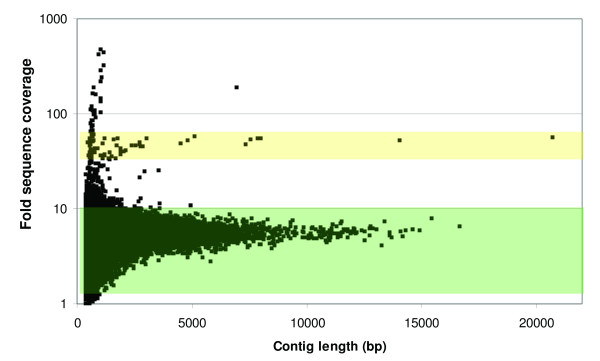
**Scatterplot of contig coverage versus length for the unfiltered assembly**. Fold sequence coverage of assembled contigs is distributed relatively narrowly around the mean, with few outliers. Contigs with coverage within the range 2-10× (shaded green) were included for further analysis as putative nuclear genomic contigs of *V. destructor *(see text for details). Short, high-coverage contigs are predominantly mitochondrial or ribosomal in nature. A cluster of longer contigs approximately with 30-50× coverage appear to derive from a baculovirus (see text); contigs in this coverage range are shaded in yellow.

We first examined the distribution of contig-mean coverage versus length (Figure 2), which revealed relatively few outliers, and these were found to be enriched in mitochondrial and ribosomal sequences. Thus, the vast majority of the assembled contigs do not appear to contain many collapsed nuclear repeats. Based on the observed median and variance of coverage (Figure 2), we excluded contigs with less than 2× coverage and greater than 10× coverage, as well as contigs less than 300 bp in length, from the analyzed *V. destructor *assembly.

**Figure 3:**
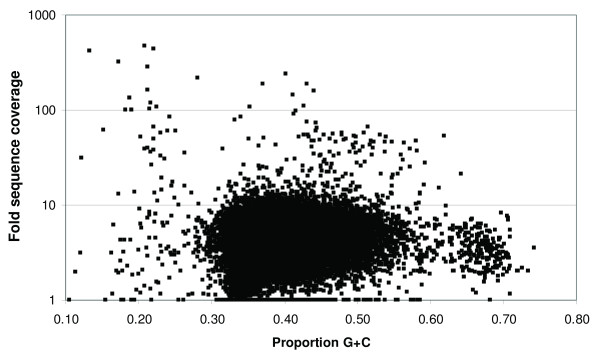
**Scatterplot of contig G+C content versus coverage**. Scatterplot of contig G+C content versus fold sequence coverage shows a clear mode of G+C content in the range of 32-56%. The long tail of low G+C contigs includes low-complexity sequences such as AT repeats, as well as mitochondrial and ribosomal contigs. A secondary mode of high G+C contigs is also apparent; these contigs include many BLAST matches to the bacterial order Actinomycetales (see text).

**Figure 4:**
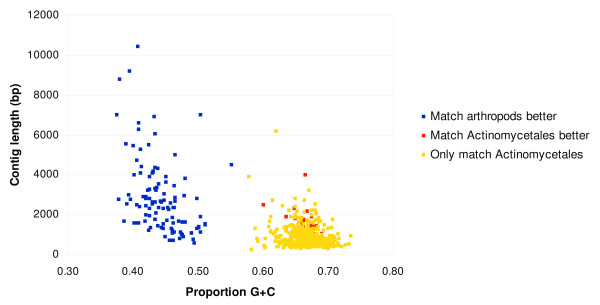
**Evidence that high G+C contigs are bacterial**. Contigs with a BLASTX match to the bacterial order Actinomycetales (at an expectation of 10^-8 ^or less), plotted as a function of G+C content and contig length. Points are color-coded according to the taxonomy of the best GenBank match overall. There is a clean separation between contigs with lower G+C that are more similar, by percent identity and expectation of BLASTX sequence alignments, to arthropod sequences and contigs with higher G+C that are more similar to actinomycete sequences.

We then examined G+C content of contigs (Figure 3), which suggested that *Varroa *nuclear DNA falls largely between 32-58% G+C content (40.9% G+C on average). Contigs with lower G+C content and higher coverage showed strong homology to mitochondrial and ribosomal DNA sequences previously reported for *V. destructor*, as would be expected. Contigs with higher G+C consistently showed higher sequence similarity to bacterial sequences than to arthropod sequences. For example, Figure 4 illustrates the distribution of contigs with BLASTX matches to the high G+C bacterial order Actinomycetales at an expectation of 10^-8^, plotted as a function of length and G+C content of contigs. Of these contigs, only those above approximately 58% G+C content were better matches (by sequence similarity and E-value) to Actinomycetales than to arthropods, or lacked an arthropod match entirely. Based on these considerations, we removed contigs outside the range of 32-58% G+C from the analyzed *V. destructor *assembly unless they contained a superior match to a eukaryotic sequence in GenBank at an expectation of 10^-8^. Additional filtering was performed as described in the Methods to specifically remove sequences from organisms that were considered potential contaminants *a priori*, such as known microbial pathogens of honey bees that are dispersed as spores.

### Actinomycete discovery

The strong secondary mode of high G+C contigs in Figure 3 and taxonomically coherent BLAST hits (Figure 4) suggests that one or more Actinomycete species were particularly abundant in the sequenced sample. Together these contigs totaled ~300 Kbp of sequence, which were further analyzed with the BLAST2GO annotation tool [34]. Additional file 1 includes BLAST2GO output that summarizes the distribution of BLASTX hits among these contigs with respect to matched species, expectation, and sequence similarity. All of the most frequently matched organisms are members of Actinomycetales. Ribosomal sequences were also found that had strong similarity to this clade, the closest match (98% identity) being to the genus *Segniliparus*.

To further investigate the distribution of this bacterium in *Varroa *mites, we designed primers specific to a homolog of translation initiation factor TIF-3. These primers amplified the target sequence from DNA of individual mites, as confirmed by sequencing of the amplicon. The rate of infection in a sample of mites (see Methods) is shown in Table 2. Rates of infection varied significantly (P < 0.01, χ2 test) by age and sex. Mature females were infected more often than males (61% versus 33%). We did not detect the infection in a small sample of eggs and it was rare in nymphs (11%). These data suggest that horizontal transmission of the bacteria occurs within capped cells (from which males do not leave). We were not able to amplify the target from *A. mellifera *DNA extracted from adult bee abdomens, either with these primers or another pair targeting an ABC transporter gene (see Methods). Given the intimacy of *Varroa *mites with their hosts, this result suggests that the bacterium has some specificity to *Varroa*. While additional surveys at broader geographical scales are needed to confirm and extend these conclusions, the amount of sequence classified as actinomycete is remarkable given that common microbial components of the arthropod gut flora [35] (e.g., the Enterobacteriaceae) were not strongly evident in the initial assembly (see Methods). However, as the mites were not surface sterilized prior to extraction of genomic DNA, it is unclear whether the infection is topical or internal.

**Table 2:** Frequency of infection of individual mites by a novel actinomycete bacterium identified in the *V. destructor *sequence survey

Class	Present	Absent
Male	4	8

Female	11	7

Nymph	2	16

Egg	0	5

Presence or absence of the bacterium is based on a PCR assay (see text for details).

### Baculovirus discovery

Curiously, the longest contig in the initial assembly was among those filtered due to high coverage across its entire length (a mean of 56×). In fact, this contig is part of a distinct group visible in Figure 2 that have mean coverage around 50×, sloping down to ~30× as contig length decreases (a frequently observed phenomenon attributable to low-coverage at contig ends). We therefore investigated whether contigs ≥ 1 Kbp in length and with 30-60× coverage were of a consistent type, such as microbial DNA or a class of repetitive sequence (e.g., ribosomal genes or transposable elements). BLASTX and Pfam [36] searches indicated that these high coverage contigs derive from a novel baculovirus. Baculoviruses are large DNA viruses that are common in certain arthropod taxa and often have strong impacts on host survival (reviewed by [37]). Known baculoviruses range from 80 to 180 kb in length and typically encode more than 100 ORFs, some of which are putatively specific to Baculoviridae. Indeed, several baculovirus-specific domains were detected among the 216 methionine-initiated ORFs greater than 90 amino-acids in length that were found on these high-coverage contigs (Table 3). Domains related to viral genome replication as well as other domains previously reported in Baculoviridae were also found. The baculovirus *Spodoptera litura *NPV appears to be the most similar viral species overall in GenBank, as there were strong matches to ribonucleotide reductase subunits RR1 and RR2 and weaker matches to a number of other peptides of this viral species. A number of ORFs encoding low-complexity proteins were identified from these high-coverage contigs, but it remains unclear which of these, if any, are viral in nature (all filtered contigs and predicted ORFs are given in Additional file 2). PCR primers specific to two separate loci (see Methods) confirmed the presence of these sequences in the original sample pool, but we were able to amplify these loci from only two of fifty individual mites collected at different times from the same colonies as the sequenced sample. Thus, the putative baculovirus-like sequences do not appear to be common in *V. destructor*, although we can infer from their high coverage in the initial assembly that high titres are sometimes achieved. This result also demonstrates that these sequences are not integrated into the *V. destructor *genome. Because baculoviruses are important tools for arthropod transgenics and biocontrol [38,39], efforts to clarify the nature of this virus and its hosts are underway.

**Table 3:** Evidence for a novel virus related to the Baculoviridae in the sequenced sample of *Varroa destructor*

ORF	Pfam domain description	Expectation	Reported in Baculoviridae?
VDK00007920-4466_1	Ribonucleotide reductase, barrel domain	5.50E-231	Yes

VDK00121146-847_1	Ribonucleotide reductase, small chain	1.30E-125	Yes

VDK00001240-6963_1	Thymidylate synthase	3.60E-114	Yes

VDK00008686-4345_1	Kinesin motor domain	3.90E-060	No*

VDK00103915-1040_1	Reverse transcriptase	7.80E-035	Yes

VDK00064516-1660_1	BRO family, N-terminal domain	1.60E-017	Yes

VDK00001041-7192_1	Chitin binding domain	1.50E-010	Yes

VDK00192648-381_1	Pacifastin inhibitor (LCMII)	3.60E-008	No

VDK00158309-530_2	Baculovirus hypothetical protein	1.10E-006	Yes

VDK00025611-2890_1	Matrixin (matrix metalloprotease)	3.80E-006	Yes

VDK00139482-672_1	Protein of unknown function (DUF666)	3.90E-006	Yes

VDK00179897-428_1	Phosphatidylinositol-specific phospolipase	5.60E-006	No*

VDK00008449-4382_2	Protein of unknown function (DUF686)	9.90E-006	Yes

VDK00099267-1099_1	Zinc knuckle (retroviral gag protein)	1.20E-004	Yes

VDK00107278-999_1	Baculovirus BRO family, N-terminal domain	1.80E-004	Yes

VDK00158309-530_3	Gamma-glutamyltranspeptidase	2.60E-004	No*

VDK00071395-1528_1	Collagen triple helix repeat	8.20E-004	No*

VDK00038309-2357_1	Collagen triple helix repeat	9.20E-004	No*

VDK00202991-345_3	Amelogenin (cell adhesion protein)	2.20E-003	No

VDK00042225-2226_1	Alpha/beta hydrolase fold	2.40E-003	No*

VDK00021332-3129_1	Phage integrase family	3.00E-003	No*

VDK00104983-1027_1	Collagen triple helix repeat	4.20E-003	No*

VDK00202991-345_2	Collagen triple helix repeat	4.20E-003	No*

VDK00043167-2196_3	Protein of unknown function (DUF686)	9.10E-003	Yes

VDK00073176-1494_3	Gamma-glutamyltranspeptidase	9.20E-003	No*

VDK00048355-2046_1	Matrixin (matrix metalloprotease)	0.01	Yes

Pfam matches are shown for methionine-initiated ORFs of 90 amino acids or more that were encoded by contigs at least 1 kb in length and with 30×-60× coverage. Collectively the matches are consistent with a viral origin and include several domains characteristic of Baculoviridae. Only matches with an expectation of 0.01 or less are shown. *Domain reported from at least one virus in Pfam [36] and Interpro [66] databases. **Present in one baculovirus accession (Q65353 of UniProt [67]) due to a retrotransposon insertion.

### V. destructor annotation and evolutionary comparison with other arthropods

We used BLASTX (for genomic contigs) and BLASTP (for ORFs of 90 residues or more) to identify genic sequences in the assembly. Sequences were initially searched against a database of five arthropod peptide predictions (*Drosophila melanogaster*, *Anopheles gambiae*, *Pediculus humanus *[a representative non-Dipteran insect], *Daphnia pulex*, and *I. scapularis*), with a minimum expectation of 10^-8^, and then secondarily against the eukaryotic Refseq database at the same expectation. In total, 13,031 contigs were identified with BLAST-detected similarity to database sequences (listed in Additional file 3). These contigs had a median length of 1,967 bp and summed to 31.3 Mbp, and represent roughly 10% of the total assembled sequence (294 Mbp after filtering). ORFs with significant Pfam domains are listed in Additional file 4 and the sequences are provided in Additional file 5.

These annotated gene fragments are necessarily an incomplete accounting of the number and type of genes in *V. destructor*, given the limitations of the assembly and lack of transcriptome data. Additional genomic resources are needed for robust gene models and are in development [19]. However, we used two approaches to infer how well represented *Varroa *protein-coding genes are in this survey. We first identified ORFs that were putatively homologous to enzymes of the glycolysis/gluconeogenesis pathway, using KEGG-annotated pathway components [40] from the mosquito, *Anopheles gambiae*, and the tick, *I. scapularis*, as BLAST queries. *I. scapularis *is the most closely related organism to *V. destructor *for which an extensively annotated genome sequence exists [41]. We identified 19 putative pathway components in our search (Table 4), whereas there are 21 members in *I. scapularis *and 23 in *A. gambiae*. A second, similar approach to assessing how well *V. destructor *genes were represented in the assembly was to query all predicted ORFs against the CEGMA set of hidden Markov models of evolutionarily conserved proteins [42]. A Hmmer [43] search found matches for 303 of the 458 protein models available, at an E-value threshold of 1.0 (matches at this level also had BLASTP matches in GenBank with E-values less than 10^-10^). The same search performed on *I. scapularis *predicted proteins found matches for 429 models. Given that ORFs are proxies for single coding exons, both approaches used here are likely to under-sample *V. destructor *coding sequences. These comparisons nonetheless suggest that a majority of the coding potential of *V. destructor *was captured in this survey, if in fragmented form. However, they also indicate that comparisons of gene-family or protein-domain abundance in *V. destructor *relative to other arthropods may be premature.

**Table 4:** The *Varroa destructor *glycolysis/gluconeogenesis pathway is well represented in the genome sequence survey

Glycolysis/gluconeogesis enzyme	KEGG ID	Annotated in *A. gambiae*	Annotated in *I. scapularis*	Closest contig match in the *V. destructor *assembly (BLASTX)	Strand	Start	Stop	E-value
6-phosphofructokinase	K00850	X	x	VDK00166959	+	274	486	6.00E-027

acetyl-CoA synthetase	K01895	X	x	VDK00052872	-	313	1554	3.00E-085

aldehyde dehydrogenase	K00128	X	x	VDK00013090	+	2385	3224	1.00E-138

dihydrolipoamide dehydrogenase	K00382	X	x	VDK00011534	-	343	917	1.00E-020

enolase	K01689	X	x	VDK00029529	-	752	2211	7.00E-143

fructose-1,6-bisphosphatase	K03841	X	x	VDK00132162	+	158	355	1.00E-023

fructose-bisphosphate aldolase	K01623	X	x	VDK00012888	+	801	2121	1.00E-073

glucose-6-phosphate isomerise	K01810	X	x	VDK00034893	+	2	145	6.00E-018

glyceraldehyde 3-phosphate dehydrogenase	K00134	X	x	VDK00020468	-	142	2477	2.00E-077

hexokinase	K00844	X	x	VDK00023511	-	313	1522	2.00E-094

phosphoenolpyruvate carboxykinase	K01596	X	x	VDK00063370	-	1166	1684	1.00E-038

phosphoglucomutase	K01835	X	x	VDK00033184	-	236	478	3.00E-024

phosphoglycerate kinase	K00927	X	x	VDK00052433	+	351	1229	4.00E-032

phosphoglycerate mutase	K01834	X	x	VDK00033074	-	257	2550	5.00E-031

pyruvate dehydrogenase E1 component, subunit alpha	K00161	X	x	VDK00079694	-	2	1382	8.00E-043

pyruvate dehydrogenase E1 component, subunit beta	K00162	X	x	VDK00041927	-	527	2110	2.00E-020

pyruvate dehydrogenase E2 component	K00627	X	x	VDK00062508	-	165	416	4.00E-033

pyruvate kinase	K00873	X	x	VDK00096436	-	676	1131	4.00E-067

S-(hydroxymethyl)glutathione dehydrogenase	K00121	X	x	VDK00003663	-	1731	2700	4.00E-125

glucose-6-phosphate 1-epimerase	K01792	X	x					

triosephosphate isomerise	K01803	X	x					

aldose 1-epimerase	K01785	X						

L-lactate dehydrogenase	K00016	X						

Putative pathway components listed below were identified by BLASTX, using KEGG-annotated components [40] from *Anopheles gambiae *and *Ixodes scapularis *as search queries.

We used RepeatMasker [44] to characterize the distribution of protein-coding transposable element classes in *V. destructor*, which are summarized in Table 5. The Mariner class of DNA transposon was by far the most abundant transposable element identified. Some retrotransposons were also common, particularly *gypsy*-type long-terminal-repeat (LTR) retrotransposons and LINEs (long interspersed nuclear elements). Helitrons, which replicate by a rolling-circle method, were also numerous. Given the quasi-clonal nature of the K and J haplotypes of *V. destructor*[23], comparative re-sequencing of these two groups could uncover recently active transposable elements. Active elements are of interest because they are important contributors to genome evolution, including the creation of novel host genes [45], and have utility for functional genomics [46].

**Table 5:** Protein-encoding transposable elements in *V. destructor*

Class	Family	Number identified
DNA	TcMar-Mariner	6511

DNA	TcMar-Tc1	357

DNA	hAT	58

DNA	TcMar-Fot1	27

DNA	MuDR	12

Helitron	Helitron	338

LINE	R1	981

LINE	L2	93

LINE	CR1	83

LINE	BovB	47

LINE	Jockey	28

LINE	L1	17

LTR	Gypsy	914

LTR	Pao	102

LTR	Copia	74

LTR	Gypsy-Cigr	31

Transposable elements identified with the protein-level repeat masking mode of RepeatMasker [44]. Class abbreviations are DNA, type II DNA transposon; LINE, long interspersed nuclear element; LTR, long-terminal repeat retrotransposon. Family designations are those of RepBase [68].

**Figure 5:**
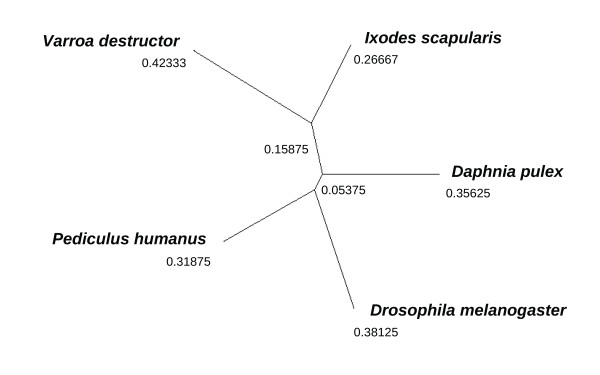
**A distance-measure dendrogram of five arthropod taxa based on aligned blocks of conserved peptides**. The unrooted tree was constructed with Phylip [64] using neighbor joining and the JTT exchange matrix as described in the text. The tree was drawn with TreeView [65].

Although the *I. scapularis *genome should be a valuable guide for homology-based gene prediction in *V. destructor*, these lineages are estimated to have diverged 336 ± 26 million years ago [47]. It is therefore of interest to assess the level of sequence divergence between these two taxa, as well as the divergence of these Acari from other model arthropods. We identified 730 peptide blocks, averaging 128 residues in length, that were conserved between putative orthologs in these two species and among their closest homologs in *Da. pulex*, *Dr. melanogaster*, and *P. humanus*. We then calculated the average genetic distance among species for each block, using the JTT substitution matrix [48] and weighted by block length (see Methods). The unrooted dendrogram derived from a total of 94,146 aligned positions is shown in Figure 5. The *V. destructor *branch (0.42333) is substantially longer than the *I. scapularis *branch (0.26667) from their shared common ancestor, implying a high rate of amino-acid evolution in the *Varroa *lineage and/or a low rate of amino-acid evolution in the *Ixodes *lineage. Note that this distance-based approach does not require specifying a nucleotide substitution model or correcting for multiple substitutions; the branch lengths are approximately proportional to the product of the time since divergence and the branch-specific rate of molecular evolution (see [49] for a discussion). Furthermore, the branching order of these taxa are well-supported by independent data [50,51]. Of course, this result is averaged across many loci and does not necessarily represent the pattern of sequence divergence at any given gene. It remains to be seen whether other gene features, such as exon structure, have also evolved at a comparably divergent rates.

### Nucleotide polymorphism in *V. destructor*

Life-history traits of *V. destructor *[5] that act to reduce genetic polymorphism within family lineages include male haploidy [25] and a predominance of full-sib mating. Genetic variation within a population can nonetheless be high in principle if populations are admixtures of distinct lineages. High-frequency polymorphisms, whether due to admixture or heterozygosity, can cause difficulties for shotgun assembly algorithms because they weaken the computational discrimination of allelic and non-allelic sequences. On the other hand, polymorphisms may be useful as genetic markers for population-genetic studies. It is therefore of interest to estimate levels of genetic polymorphism in the sequenced sample (~1,000 mites drawn from three adjacent colonies). We used the program SWAP454 [52] to estimate the occurrence of moderate- to high-frequency SNPs, i.e. those present in reads at a minimum ratio of 0.1 to the assembly reference base. SNP calls also required a minimum of two reads with the alternative base, but did not require reads in both directions. Polymorphisms meeting these parameters occurred at a rate of 6.2 × 10^-5 ^per base pair. Given a median per-contig coverage of 5.0× in the final assembly (Table 1), our ability to detect low frequency polymorphisms is of course limited, but such polymorphisms contribute much less ambiguity to genome assembly.

To further investigate the potential for sequence polymorphism within a *V. destructor *population, we identified trinucleotide microsatellite loci in the genomic sequence and obtained amplification products for ten of these (see Methods). Consistent with our estimate of SNP frequency, we found no polymorphism at these loci in 65 individual mites collected from research apiaries at the USDA-ARS facility in Beltsville, Maryland, the source of the genome survey pool.

## Discussion

*V. destructor *is considered the most damaging honey-bee pest and has become widespread since its host shift from *A. cerana *less than a century ago. Resistance to common acaricides has already appeared [53], and the development of new control strategies are hampered by our limited knowledge of the *V. destructor *- *A. mellifera *interaction, particularly at the genetic level. The present genome survey makes available a large number of genic sequences for analysis and manipulation by the community of researchers. The contigs we obtained from low-coverage shotgun sequencing were short, as expected, such that few complete gene models are likely to be annotated from this data set. Nonetheless, our assembly enables the identification of genes of interest and the cloning of complete transcripts as they are needed. The assembly will also greatly assist the validation and annotation of transcriptome surveys and can support proteomic initiatives. We hope the resources provided here will aid investigators already tackling the problem of mite control with molecular methods as well as encourage the involvement of others.

Genetic approaches to the study of mite control are promising for a number of reasons. Successful reproduction of *Varroa *mites requires precise coordination with the care of honey bee brood and a sophisticated evasion of honey bee defences. Chemosensory genes are among those likely to play crucial roles in this process. At the phenotypic level, there is known variation among *Varroa *haplotype groups and species in their ability to successfully parasitize *A. mellifera *[16,17], as well as known variation in the resistance of honey bee strains [14,15,54,55]. While *Varroa *mites are not tractable to controlled crosses, associative mapping of traits such as reproductive success on different hosts remains an attractive possibility, as is the mapping of resistance traits in honey bee. Resequencing efforts are needed to uncover genetic variation that can be exploited for these purposes. Those efforts would also contribute to a better understanding of the demographic history of *V. destructor *and to species relationships within the genus. Polymorphic markers within haplotype groups would aid investigations of the population biology of the species, particularly in light of the difficulty of observing or manipulating *Varroa *mites in their habitat. For example, estimates of outcrossing and migration might be relevant to the design of new mitigation strategies, particularly if the evolution of resistance traits is expected. While consistent with previous work [23] that found low genetic polymorphism within the predominant *V. destructor *lineage, our analysis nonetheless enables genome-scale mining of markers for population-genetic studies.

Our analysis of conserved peptide blocks showed a higher rate of protein evolution within the *Varroa *lineage relative to *Ixodes*. It remains to be clarified whether this level of sequence divergence is characteristic of mites or peculiar to the *Varroa *lineage. Similarly, whether this result correlates with divergence in other aspects of genome evolution, such as exon structure and regulatory features, will be an important question to pursue as annotations improve for both species. As genomic resources for *V. destructor *continue to improve, opportunities for evolutionary comparisons with other arthropods will be enriched. Such comparisons are of tremendous value because they can expose conserved elements that might otherwise elude detection by direct experiment, and they reveal the relative rates at which various classes of homologous sequence diverge. *Varroa *mites and others in the Parasitiformes comprise a lineage that diverged from ixodid ticks over 300 million years ago [56], while the chelicerates as a whole branched from the insects and crustacea 725 million years ago [57]. Consequently, as a representative of the Parasitiformes, *Varroa *provides a key evolutionary landmark for comparative studies across arthropods currently targeted for genomic analyses.

An accessory goal of genome projects targeting arthropod pests is the identification of novel microbes and viruses that may be relevant to the epidemiology of vectored diseases, or that lend themselves to biocontrol programs. A significant finding of this study was the discovery of an actinomycete bacterium that infects *V. destructor *at intermediate frequencies (albeit presumably at high titer given its abundance in the genome sequence) but apparently does not infect *A. mellifera *at appreciable levels. However, these findings are preliminary and await a more systematic survey of infection among mites and bees. Future research should also be directed toward isolating this bacterium and assessing the fitness consequences of infection. Further characterization of the putative baculovirus identified in this survey is similarly a priority.

This work contributes to the relatively small body of genomic studies to date that have applied next-generation sequencing to a complex eukaryotic genome phylogenetically distant from other reference genomes. As the costs and technical requirements for genome sequencing continue to decline, such studies will undoubtedly become commonplace. In many cases, the sequencing of a single genotype or inbred group will not be feasible, and there may also be a significant metagenomic contribution from associated microbes. While these factors introduce new challenges, our results underscore the utility of these methods for rapidly advancing the study of non-model organisms.

## Conclusions

Our results have provided general tools for the research community and novel directions for investigating the biology and control of *Varroa *mites. Ongoing development of *Varroa *genomic resources will be a boon for comparative genomics of under-represented arthropods, and will further enhance the honey bee and its associated pathogens as a model system for studying host-pathogen interactions.

## Methods

### Genome-size estimation

Samples were prepared for flow cytometry as previously described [27]. For each replicate, the synganglion of a mature female *V. destructor *was placed along with the head of a mature female *D. virilis *into a 2 ml tissue grinder (Kimble-Kontes) containing 1 ml of cold Galbraith buffer, and stroked 15 times with the A pestle to release nuclei. The preparation was filtered through 20 μm nylon and stained with prodidium iodide to a final concentration of 50 ppm. Stained samples were held on ice in the dark for 1-2 hr prior to analysis. The mean fluorescence of stained nuclei in replicate preparations of *Varroa *and *D. virilis *standard was quantified using a Coulter Epics Elite flow cytometer (Coulter Electronic), with excitation provided by a laser tuned at 488 nm and 25 mW. PI fluorescence at > 615 nm was detected by a photomultiplier screened by a long pass filter. To ensure that scoring included only intact nuclei free from cytoplasmic tags, counting was activated by red fluorescence (discrimination), and only (gated) nuclei with low forward and side scatter were included in the analysis. The positions of sample peaks relative to the *D. virilis *peak were verified by running samples without a standard. DNA content was determined from co-preparations as the ratio of the 2C *Varroa *peak to the 2C *D. virilis *peak times the 1C genome size of *D. virilis *(333 Mb, after [58]).

### Sample preparation and sequencing

*V. destructor *mites were collected on two occasions for sequencing from infested colonies of the USDA-ARS Bee Research Laboratory apiaries in Beltsville, MD, USA in October, 2008. For each collection, 300 bees were placed into a 0.5 liter glass jar containing 40 g confectioner's sugar. The sugar and bees were thoroughly mixed by shaking the jar for 30 s. The sugar and phoretic mites were then separated from their bee hosts by shaking the jar contents through a 1 mm wire mesh placed at the jar opening. Mites were shaken into a small water bath, which was then poured through a cheesecloth filter and rinsed twice with sterile water to remove residual sugar. Live mites were then picked onto sterile tissue paper and frozen at -80°C until nucleic acid extraction.

To obtain sufficient high-quality DNA for six pyrosequencing runs, three separate DNA extractions were made from the collected mites. DNA from one sample of ~400 mites was extracted with DNAzol (Invitrogen) following the manufacturer's instructions. A second sample of ~400 mites was homogenized in 800 μl proteinase K buffer (10 mM NaCl, 10 mM Tris, 50 mM ethylenediamenetetracetic acid (EDTA), and 10 μg/ul proteinase K) and incubated 60 min at 55°C, vortexing every 20 min. Afterwards, 180 μl of 8 M potassium acetate was added and the sample incubated on ice for 30 min. After high-speed centrifugation, DNA was precipitated from the supernatant with ethanol and re-suspended in distilled, deionized water. A third sample of ~200 mites was homogenized in 500 μl hexadecyltrimethylammonuim bromide (CTAB) buffer (100 mM Tris-HCl at pH 8.0, 20 mM EDTA, 1.4 M NaCl, 2% CTAB, and 0.2% β-mercaptoethanol) and incubated 60 min at 65°C, vortexing every 20 min. An equal volume of 24:1 chloroform:isoamyl alcohol was then added and the sample centrifuged at high speed. DNA was precipitated from the supernatant with isopropanol and re-suspended in distilled, deionized water. For all extractions, DNA concentration and quality were evaluated with a Nanodrop ND-8000 spectrophotometer and were found to be comparable. DNA quality was also checked by gel electrophoresis.

Pyrosequencing was performed at the Institute for Genome Sciences, University of Maryland School of Medicine, on a Genome Sequencer FLX instrument (454 LifeSciences) using GS-FLX titanium reagents. DNA was prepared for emulsion PCR according to the manufacturer's protocols.

### Assembly and analysis

Contigs were assembled with the CABOG package of Celera Assembler version 5.2 [31]. The sequences were assembled iteratively, adjusting the assumed error rate incrementally between 0% and 6%. The assembly selected for analysis used a 1.5% error rate because this value maximized the length of the longest contig (18.7 Kbp). The weighted median N50 contig size was relatively stable across iterations at ~2.1 Kbp, meaning that half of the assembled bases were consistently contained in contigs of this size or larger across the different assemblies.

Contigs were screened to identify sequences of organisms that were considered potential contaminants *a priori*. For example, a number of contigs were found to be nearly identical to the honey bee reference genome [59]. These fragments included low-copy genic sequences as well as ribosomal, nongenic, and mitochondrial sequence. PCR primers specific to *A. mellifera *sequences amplified genomic DNA extracted from adult female mites but not from embryos (data not shown), implying that the source of *A. mellifera *contamination is bee hemolymph consumed by mites. In contrast, searches against the genome sequences [21] of chalkbrood (*Ascosphaera apis*), a fungal pathogen of honey bees, and American foulbrood (*Paenibacillus larvae*), a bacterial pathogen, did not reveal the presence of these spore-dispersed microbes in the sample.

All contigs were also screened for general bacterial contamination by searching ORFs of 90 codons or more against the RAST [60] seed database of bacterial sequences. ORFs were identified with the *getorf *program of the EMBOSS package [61]. ORFs with significant matches to the RAST database were then searched by BLASTP against the full GenBank nr database to determine the most closely matching organism. Candidate microbial sequences are summarized in Table 6. As expected (see Results), matches to the phylum Actinobacteria are the vast majority. Among the small number of BLASTP matches to other bacterial groups, the genera *Burkholderia *(β-proteobacteria) and *Pseudomonas *(γ-proteobacteria) were the most represented taxa.

**Table 6:** Distribution of contigs that were designated bacterial by BLAST analysis, sorted by phylum

Phylum	Number of contigs
Actinomycete	1035

α-proteobacteria	10

Aquificae	1

Bacteroides	2

β-proteobacteria	13

Cyanobacteria	3

δ-proteobacteria	3

Euryarachaeota	1

Firmicutes	4

γ-proteobacteria	12

Because many organisms show distinct patterns of codon usage [62], we compared codon usage for ORFs from unfiltered contigs (putatively *V. destructor*) with those from putatively bacterial contigs. Only ORFs with BLAST-supported homology to GenBank sequences were used for this comparison. We used the program INCA [63] to plot the codon-usage statistic 'B' of [62] as a function of third-position G+C (GC3) content (Additional file 6). The value of B for a given ORF is a measure of how similar its codon usage is to the overall codon usage in the data set. GC3 is considered here because third positions are much less constrained by protein function than first and second positions, and thus more indicative of background composition biases. The distinct patterns observed for the two groups of ORFs provide complementary evidence that these sequences do in fact derive from different organisms. The plot also shows that a few ORFs from contigs considered to be *Varroa *by our filtering methods may in fact be bacterial in nature and merit further evaluation. Of course, BLAST-supported ORFs are only proxies for transcripts and thus individual points may be highly inaccurate. In general, however, genic sequences that are putatively from *V. destructor *have a cohesive pattern of codon usage that can be distinguished form at least some bacterial contaminants, regardless of detectable homology.

To confirm the presence in mites of the actinomycete bacterium and DNA virus identified in the assembly, individual eggs, nymphs, female adults, and male adults were collected from parasitized honey bee pupae. DNA for PCR was extracted from individual samples by grinding them in 200 μl of 5% Chelex-100 solution (Bio-Rad), incubating at 65°C for 30 min, pelleting the mixture by high-speed centrifugation, and taking a 1:10 dilution in water of the resulting supernatant. Primers for the putative actinomycete TIF3 locus were CCGATCTCGACCTTGTGGAA (forward) and CTCGGAACATGATCGTCACC (reverse), and for the ABC locus were GAGGTCCTCGTCTCCGAATG (forward) and CGATGTCCTGGATCCTCTGG (reverse). The amplified TIF3 product was confirmed by Sanger sequencing (GenBank:GU365869). Primers designed to amplify the putative Baculovirus targeted a ribonucleotide reductase small subunit gene (forward ACGAACGACTATCTAGCCATGAAC and reverse GTCCGTTTCGGAGTGCATGAC) and a thymidylate synthase gene (forward CGCATGTACCAACAACTCGTAC and reverse CACAGTTGGTGTAGCGCAGT). The identities of these products were also confirmed by Sanger sequencing (GenBank:GU980896 and GenBank:GU980897, respectively). All PCR reactions were performed using standard reagents and thermocycler protocols, with an annealing temperature of 54°C.

To identify conserved peptide blocks, we first identified *V. destructor *ORFs that were reciprocal best BLASTP matches with *I. scapularis *predicted peptides. These were then used to identify the closest homologs in *Da. pulex*, *Dr. melanogaster*, and *P. humanus*. Sequences were aligned by ClustalW and then trimmed to include only blocks of well conserved, unambiguously aligned sequence for which we could have reasonable confidence of orthology. Genetic-distance matrices were calculated for each block with the *protdist *program of the PHYLIP package [64], weighted by alignment length and summed, then normalized to a maximum distance of one. Unrooted dendrograms were constructed with the fitch and neighbor programs of the PHYLIP package, giving virtually identical branch lengths; the neighbor-joining dendrogram is shown in Figure 5.

Microsatellite loci used to assess polymorphism levels in *V. destructor *are characterized in Additional file 7. PCR amplifications consisted of 1 U Taq DNA polymerase with appropriate buffer, 1 mM dNTP, 2 mM MgCl2, 0.2 μM of each forward and reverse primer in a final reaction volume of 5 μl. Fluorescently labeled primers were mixed with unlabeled primers at a 12:20 ratio. Thermocycling was performed as follows: 96°C for 2 min., then 3 cycles of 96°C for 30 sec., 60°C for 30 sec. (-1°C/Cycle), 65°C for 1 min., followed by 35 cycles of 96°C for 30 sec., 56°C for 30 sec., 65°C for 1 min, and a final extension at 65°C for 2 min. PCR products were diluted 1:20 and 1 μl of this dilution was added to 10 μL formamide containing the LIZ size standard. Products were analyzed by capillary electrophoresis using an Applied Biosystems 3730XL instrument. Allele sizes were scored using ABI GeneMapper version 3.7 (Applied Biosystems).

## Authors' contributions

RSC led the bioinformatic analyses and writing. JDE designed and coordinated the project and helped with analyses and writing. MCS generated the genome assembly. JSS performed the genome size estimation. YPC and JSP participated in project design and the collection of biological materials. GH, LB, CE, DA, and CMG aided in data analysis and manuscript preparation. All authors read and approved the final manuscript.

## Supplementary Material

Additional file 1 **Annotation statistics for filtered high G+C contigs**. Annotation statistics derived from the BLAST2GO annotation tool [34]. A. Distribution of BLASTX hits (E ≤ 10^-10^) by organism. Note that the "Others" category is typically the most abundant in this type of analysis because of the wide taxonomic distributions of many conserved proteins. B. Distribution of BLASTX hits by sequence similarity score. C. Distribution of BLASTX hits by expectation. Click here for file

Additional file 2 **Contigs identified as deriving from a novel virus of the Baculoviridae**. Fasta-formatted contigs were classified as described in text. Also included are the methionine-initiated ORFs of 90 codons or more that are referenced in Table 3. Click here for file

Additional file 3 **BLAST-annotated *Varroa *contigs**. Spreadsheet of *Varroa destructor *genomic contigs with significant similarity to the GenBank nr database by BLAST search. Click here for file

Additional file 4 **ORFs with Pfam domains**. Spreadsheet of significant Pfam domains within ORFs having BLASTP-detected similarity to GenBank sequences. Click here for file

Additional file 5 **ORFs referenced in Additional file **3**and Additonal file **4. Fasta-formatted ORFs from *V. destructor *contigs that had significant sequence similarity to database sequences. Click here for file

Additional file 6 **Comparison of ORF codon usage for contigs assigned as either *Varroa destructor *or bacterial in origin**. Scatterplot comparing G+C content and codon usage of ORFs from contigs assigned as either *Varroa destructor *or bacterial. The X-axis value is third position G+C and the Y-axis value is the codon usage statistic B [62], which as used here is a measure of the difference in codon usage between each subgroup of ORFs relative to the whole. The possible range of values for B is 0 to 2, with larger values corresponding to greater divergence in codon frequencies. A. Residual contigs after filtering, which are assumed to all derive from *V. destructor*. B. Contigs filtered because they have a higher BLAST score to bacterial sequences than to eukaryotic sequences. The two groups of contigs have distinct patterns of codon usage and nucleotide composition. Click here for file

Additional file 7 **Microsatellite loci used in *Varroa destructor *polymorphism survey**. Table lists contig containing the microsatellite locus, forward and reverse primer sequences, expected product size based on the reference contig, and the 5' start coordinate on the contig for the forward primer. Click here for file
