# Supplementary figures and images for: Genomic survey of the ectoparasitic mite Varroa destructor, a major pest of the honey bee Apis mellifera
Source: BMC Genomics. 2010 Oct 25;11:602. doi: 10.1186/1471-2164-11-602 (PMC3091747; doi:10.1186/1471-2164-11-602)

A

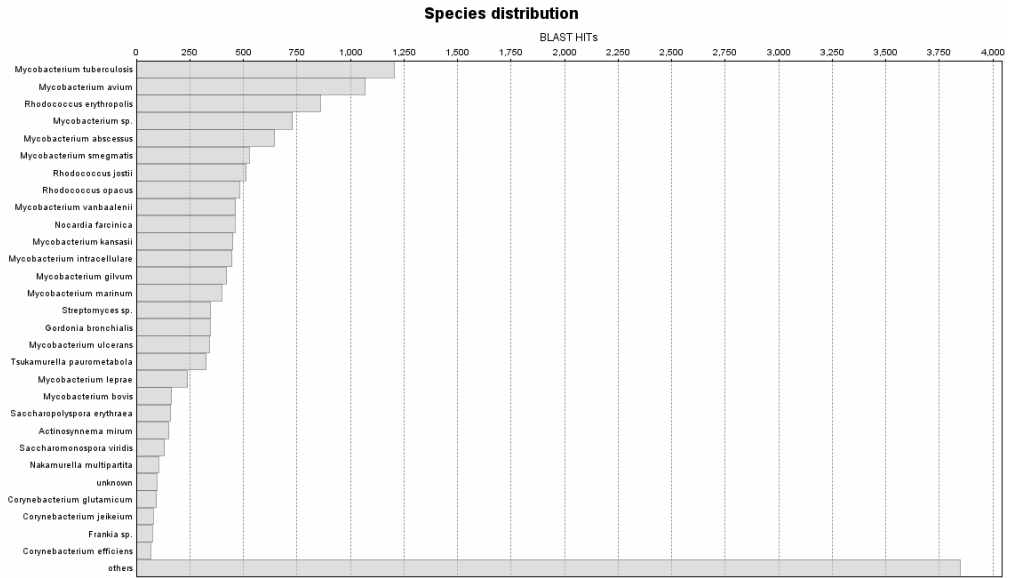

B

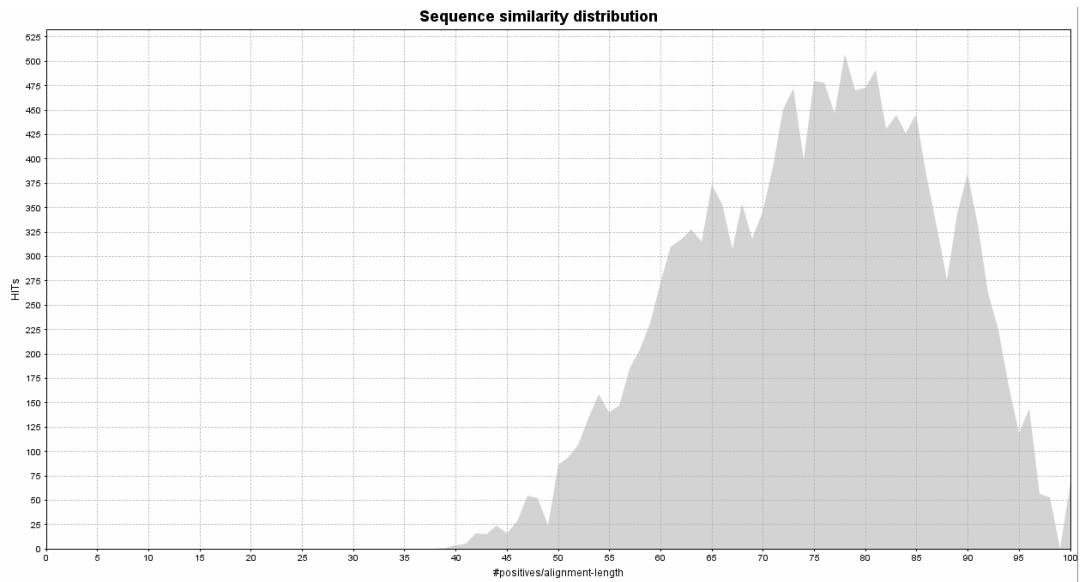

C

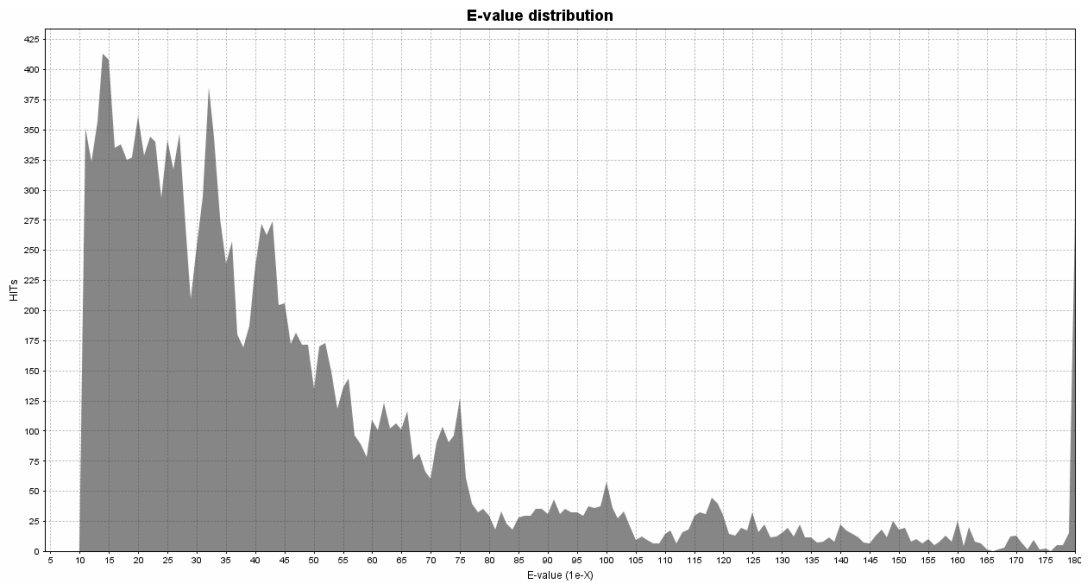

Supplement: Additional file 1 — Annotation statistics for filtered high G+C contigs. Annotation statistics derived from the BLAST2GO annotation tool [34]. A. Distribution of BLASTX hits (E ≤ 10-10) by organism. Note that the "Others" category is typically the most abundant in this type of analysis because of the wide taxonomic distributions of many conserved proteins. B. Distribution of BLASTX hits by sequence similarity score. C. Distribution of BLASTX hits by expectation. [file 1471-2164-11-602-S1.PDF]
