# Supplementary material for: Genomic survey of the ectoparasitic mite Varroa destructor, a major pest of the honey bee Apis mellifera
Source: BMC Genomics. 2010 Oct 25;11:602. doi: 10.1186/1471-2164-11-602 (PMC3091747; doi:10.1186/1471-2164-11-602)
Supplement: Additional file 7 — Microsatellite loci used in Varroa destructor polymorphism survey. Table lists contig containing the microsatellite locus, forward and reverse primer sequences, expected product size based on the reference contig, and the 5' start coordinate on the contig for the forward primer. [file 1471-2164-11-602-S7.PDF]

Additional file 7. Table lists contig containing the microsatellite locus, forward and reverse primer sequences, expected product size based on the reference contig, and the 5’ start coordinate on the contig for the forward primer.

Contig	Forward	Reverse	Size	5' start
**VDK00002218-6180**	CCGTAATTCGTTCGCAGTTTAT	GGAGGGTAGTTTTGCCGTAAG	373	4405
**VDK00018134-3348**	GCTTGCGATTTTGTATGTCAGT	CGTTTATGTGTGATTTTGGGTC	330	3279
**VDK00027405-2801**	TTAAGCACTTTACTGCGTCTGC	TACGCCGGTATATTTAAGGCAC	181	188
**VDK00041885-2236**	AGTATAGGATGAGAAGGCGGAT	TAGTGCGAATAGCGACCAG	386	1338
**VDK00057620-1811**	ATCAGCGAAAAGAAAGGGGA	CGCAATAACGTAAACAGCACAT	286	49
**VDK00059601-1767**	ACGGTATCCACTGCGTGACT	ATCATCAGTTGTTGCTCACCAC	127	1584
**VDK00068111-1590**	TGGTTGATGACCAGAGTTTACG	CTCGTCCTTGTTGCTATTGTTG	117	742
**VDK00078546-1402**	CTAATACCACCAAATGCTTCCG	CACAACAACAACAACAACAACG	162	905
**VDK00090458-1222**	GGTCAATGGGTAACTGGGTAGA	TGAGTAGTGAGAGTCGTCGTCC	253	880
**VDK00092706-1188**	CTATCCCGAACTACTGCCTTTG	TCTTTTACCTTGTTTCTGCGTG	194	975
